# Factors associated with prior testing for HIV, Syphilis, and Hepatitis B and C among transgender women and travestis in Brazil

**DOI:** 10.1590/1980-549720240008.supl.1

**Published:** 2024-08-19

**Authors:** Beo Oliveira Leite, Inês Dourado, Laio Magno, Sandro Sperandei, Carla Gianna Luppi, Maria Amelia de Sousa Mascena Veras

**Affiliations:** I Universidade Federal da Bahia, Institute of Public Health – Salvador (BA), Brazil.; II Universidade do Estado da Bahia, Life Sciences Department – Salvador (BA), Brazil.; III Western Sydney University, Translational Health Research Institute – Penrith, Astralia.; IV Universidade Federal de São Paulo, Paulista School of Medicine, Preventive Medicine Department – São Paulo (SP), Brazil.; V Santa Casa de São Paulo, School of Medical Sciences – São Paulo (SP), Brazil.

**Keywords:** Transgender persons, Serologic tests, HIV, Syphilis, Hepatitis B, Hepatitis C

## Abstract

**Objective:**

To investigate the prior testing for HIV, syphilis, hepatitis B (HBV), and hepatitis C (HCV) among transgender women and *travestis* (TGW) in five Brazilian cities and identify factors associated with each of these previous tests.

**Methods:**

This is a cross-sectional study with the recruitment of TGW through respondent-driven sampling (TransOdara Study). The investigated outcome variable was prior testing for HIV, syphilis, HBV, and HCV in the last 12 months. The association between sociodemographic and behavioral factors with the outcome was analyzed using a binomial logistic regression with mixed effects. Adjusted odds ratios (aOR) and 95% confidence intervals (CI95%) were estimated.

**Results:**

The proportions of individuals with prior testing in the past year were as follows: 56.3% for HIV, 58.0% for syphilis, 42.1% for HBV, and 44.7% for HCV. Negative associations with prior testing were observed for individuals aged 35 years or older, whereas positive associations were found for those with high school education, those who experienced verbal or psychological violence in the last 12 months, and those who had commercial or casual partners in the last 6 months.

**Conclusion:**

There was low frequency of testing in the 12 months preceding the study for HIV, syphilis, HBV, and HCV compared to the guidelines established by the Ministry of Health. Expanding access to and engagement with healthcare and prevention services for TGW is an essential strategy in reducing the transmission chain of HIV and other sexually transmitted infections (STIs).

## INTRODUCTION

It is estimated that, around the world, about one million new sexually transmitted infections (STIs) occur every day in the general population. Most are asymptomatic and, when undiagnosed and not treated in time, may have a negative impact on individual health, as well as more direct expenses in health^1^. Therefore, timely testing is essential for epidemiological surveillance actions, that is, the identification and treatment of new cases and, consequently, interruption of the chain of transmission.

Among the several strategies proposed by the World Health Organization (WHO) to prevent STIs from growing, integrated multiple testing consists of the investigation of infections through the combined offer of several rapid tests in the same visit (e.g., HIV, syphilis, viral hepatitis etc.) for diagnosis to take place in a timely manner, connection of individuals with the service, immediate treatment, and promotion of prevention actions. For such actions to occur, the expansion of testing access for different STIs must be a priority aligned with actions of prevention and health promotion^1^. However, there are failures in access and loss of opportunities for testing, which is disproportionally higher among groups that are more vulnerable to these infections^2,3^.

For these population groups, also known as key populations (e.g., injectable drug users, prisoners and other individuals in custody, sex workers, men who have sex with other men and transgender people), it is possible to observe higher risk and vulnerability regarding STI infection, especially when higher prevalence rates are observed for these infections in comparison to the general population^3-5^. Besides, there is less access to health services addressed to prevention, testing and treatment^3-10^.

Among trangender people, transgender women and *travestis* (TGW) all over the world are disproportionally more affected by STIs. The global estimated prevalence of HIV for transgender women is 19.9%^11^; of syphilis, between 1.4 and 50.4%^12^; and the estimated prevalence rates of hepatitis B (HBV) and C (HCV) are 5.0 and 6.0%, respectively^13^. In Brazil, prevalence rates are disproportionally higher in comparison to the general population^4,14^: HIV, ranging from 14.3 to 40.9%^11,13^; syphilis, from 28.9 to 61.7%^12-17; HBV, from 0.7 to 12,3%16,18,19^; and HCV, from 0.8 to 1.4%^16,18,19^.

The additional risk and vulnerability for STIs among TGW can be explained by several factors: behavioral ones, such as sex without protection; programmatic, such as lower access to health services; and social, such as stigma and gender-based discrimination, to which this population is usually submitted^20,21^. Besides, understanding the intersection between these multiple factors is essential to assess the different levels of risk of HIV as a complex phenomenon. The investigation of these factors is important to increase knowledge about the set of aspects that characterize the vulnerability of these populations and to promote improved access to STI testing. The objective of this study was to investigate and conduction of prior testing for HIV, syphilis, hepatitis B (HBV) and C (HCV) among TGW in five Brazilian cities, and to identify the factors associated with testing.

## METHODS

### Design, location and study population

This analysis is part of the TransOdara study, called “Study of prevalence of syphilis and other STIs among transgender women and *travestis* in Brazil: care and prevention”, carried out between December 2019 and July 2021 in five capitals (Campo Grande, Manaus, Porto Alegre, Salvador and São Paulo)> The study was coordinated by the School of Medical Sciences of Santa Casa de São Paulo, in collaboration with the Center of Reference and Training in STD/Aids (CRT DST/Aids), Universidade Federal do Rio Grande do Sul (UFRGS), Universidade Federal da Bahia (UFBA), Universidade Federal do Mato Grosso do Sul (UFMS), Fundação Leônidas e Maria Deane (Fiocruz – Manaus), Municipal Secretariat of Health of Porto Alegre (SMS-POA), Instituto Adolfo Lutz (IAL) and Universidade Federal de Ciências da Saúde de Porto Alegre (UFCSPA).

The study population was comprised of people who self-identified as transgender women and *travestis* (TGW). Trangender is a Latin American is an ethnocultural term used by some transgender people as a feminine identity.

The TGW who were eligible for this study were those who presented the following criteria:

1. Being 18 years old or older;
2. Having been nominated as male at birth, and current self-identified as female gender;
3. Living in the metropolitan area of one of the study cities; and
4. Having a valid coupon to participate in the study.

### Data collection and sampling

We used the respondent driven sampling (RDS) technique, indicated for populations considered to be difficult to access, in which recruitment has longer range when performed by participants themselves, using statistical methods to adjust the recruitment performed by peers in a contact network^22-24^.

The first participants, called “seeds”, were non-randomly selected by the researchers after formative qualitative research, in order to represent the heterogeneity of the TGW population, according to demographic and socioeconomic conditions. Each one of the seeds received up to six coupons to recruit other transgender women and *travestis* in their contact network, and so on. Each participant received two types of financial compensation: first for participating in the study, and then for each TGW recruited.

The study took place in the research centers of each one of the collaborating institutions of the five sites. Participants were informed about all of the research procedures, received educational resources, condoms and lubricant gel, confirmed the eligibility for the study and signed the informed consent form. After signing the form, participants answered a sociobehavioral survey with a previously trained interviewer. The survey was divided in questions with the following topics: socioeconomic and demographic variables; access and use of health services; use of hormones and body changes; knowledge about STIs, testing and sexual behavior; stigma, discrimination and violence; use of alcohol and other drugs. For more information, see Veras et al.^25^.

### Study variables

*Outcomes:* performance of any types of testing for STIs in the past 12 months:

1. HIV testing in the past 12 months (no vs. yes);
2. Syphilis testing in the past 12 months (no vs. yes);
3. HBV testing in the past 12 months (no vs. yes);
4. HCV testing in the past 12 months (no vs. yes).

*Predictor variables:* change in legal name (no vs. yes); age (up to 35 years old vs. older than 35); race/skin color (white, black/brown and other); schooling (elementar school, high school and higher education/more); type of household (own property, rental and unstable [for instance, temporary, not characterized as a rental]); income (less than a minimum wage, one to two minimum wages, two to three minimum wages, and three or more minimum wages); gender-based discrimination (no vs. yes); history of verbal or psychological violence in the past 12 months (no vs. yes); history of physical violence in the past 12 months (no vs. yes); history of sexual abuse in the past 12 months (no vs. yes); sex labor (no vs. yes), steady partner in the past six months (no vs. yes); casual partner in the past six months (no vs. yes); commercial partner in the past six months (no vs. yes).

### Data analysis

The variables were described as absolute and relative frequencies. The association of independent variables and each outcome was assessed using mixed logistic regression models with random intercepts for the city where data were collected (Campo Grande, Manaus, Porto Alegre, Salvador and São Paulo, representing the five Brazilian macroregions). The weight from the RDS was not used, according to the recommendation by Sperandei et al.^26^. The final multiple model was selected using the StatisticalModels package for the R language, through a *backward stepwise*, beginning with the full model and all the variables, removing each variable one at a time, until a smaller significant model be reached. The maximum likelihood (ML) was used in model selection, and restricted maximum likelihood (REML) was used in the final model adjustment. The logistic model results were presented as odds ratio (OR), and their respective 95% confidence intervals. The variabilities of each one of the outcomes were estimated using the intraclass correlation coefficient (ICC). All of the analyses were conducted in R, version 4.2.3^27^.

### Ethical aspects

The project was approved by the Research Ethics Committee of the Santa Casa de Misericórdia de São Paulo (CAAE 05585518.7.0000.5479; opinion n°: 3.126.815 – 30/01/2019), as well as by other participating institutions.

## RESULTS

Of the 1,317 interviewed TGW, 1,277 answered questions about prior testing for STIs, classified as the outcome, and were included in this analysis. Of these, 55.3% (n=706; 95%CI 52.5–58.4) had been tested for HIV in the past 12 months; 56.1% (n=716; 95%CI 53.3–58.8), for syphilis; 40.0% (n=511; 95%CI 37.3–42.8), for HBV; and 42.6% (n=544; 95%CI 39.9–45.4), for HCV (Table 1). Of the total, 30.6% (n=391) lived or worked in São Paulo; 25.3% (n=323), in Manaus; 15.5% (n=198), in Salvador; 14.8% (n=189), in Porto Alegre; and 13.8% (n=176), in Campo Grande. Most of them had not changed their legal names (70.6%), were aged up to 34 years (63.0%), were black or brown (70.1%), had studied until high school (69.9%), lived in a rented house (36.7%) or in an unstable household (36.9%) and had income lower than one minimum wage (43.3%) (Table 2).

**Table 1:** Sociodemographic and behavioral description of TGW in the TransOdara Project, 2020–2021.

Variables (n=1,277)	City	n (%)	95%CI
HIV testing in the past 12 months	São Paulo	308 (76.4)	71.6–81.2
	Porto Alegre	84 (43.8)	33.0–54.6
	Salvador	101 (50.0)	40.1–59.9
	Manaus	141 (41.6)	33.4–49.8
	Campo Grande	89 (49.2)	38.7–59.7
	General	706 (55.3)	52.5–58.4
Syphilis testing in the past 12 months	São Paulo	316 (78.4)	73.8–83.0
	Porto Alegre	88 (45.8)	35.2–56.4
	Salvador	96 (47.5)	37.4–57.6
	Manaus	146 (43.1)	35.0–51.2
	Campo Grande	88 (48.6)	38.0–59.2
	General	716 (56.1)	53.3–58.8
HBV testing in the past 12 months	São Paulo	301 (74.7)	69.8–79.6
	Porto Alegre	67 (34.6)	23.3–46.5
	Salvador	49 (24.3)	12.0–36.6
	Manaus	48 (14.2)	4.1–24.3
	Campo Grande	58 (32.0)	19.7–44.3
	General	511 (40.0)	37.3–42.8
HCV testing in the past 12 months	São Paulo	314 (77.9)	73.3–82.5
	Porto Alegre	73 (38.0)	26.7–49.3
	Salvador	54 (26.7)	14.6–38.8
	Manaus	61 (18.0)	8.2–27.8
	Campo Grande	53 (29.3)	16.8–41.8
	Geral	544 (42.6)	39.9–45.4

TGW: transgender women and *travestis*; HBV: hepatitis B; HCV: hepatitis C.

**Table 2:** Sociodemographic and behavioral description of TGW in TransOdara Project, 2020–2021.

Variables (n=1277)	n (%)	São Paulo	Porto Alegre	Salvador	Manaus	Campo Grande
Has changed legal name						
No	902 (70.6)	220 (56.3)	107 (56.6)	146 (73.7)	296 (91.6)	133 (75.6)
Yes	374 (29.3)	170 (43.5)	82 (43.4)	52 (26.3)	27 (8.4)	43 (24.4)
Age (years)						
Up to 34	804 (63.0)	214 (54.7)	115 (60.8)	141 (71.2)	218 (67.5)	116 (65.9)
35 or older	473 (37.0)	177 (45.3)	74 (39.2)	57 (28.8)	105 (32.5)	60 (34.1)
Race/skin color						
White	328 (25.7)	105 (26.9)	90 (47.6)	21 (10.6)	59 (18.3)	53 (30.1)
Black/brown	895 (70.1)	274 (70.1)	95 (50.3)	171 (86.4)	242 (74.9)	113 (64.2)
Other	44 (3.4)	11 (2.8)	4 (2.1)	6 (3)	20 (6.2)	3 (1.7)
Schooling						
Elementary school	323 (25.3)	95 (24.3)	52 (27.5)	53 (26.8)	75 (23.2)	48 (27.3)
High school	892 (69.9)	274 (70.1)	126 (66.7)	140 (70.7)	230 (71.2)	122 (69.3)
Higher education or more	62 (4.9)	22 (5.6)	11 (5.8)	5 (2.5)	18 (5.6)	6 (3.4)
Household						
Own household	337 (26.4)	111 (28.4)	62 (32.8)	70 (35.4)	51 (15.8)	43 (24.4)
Rental	469 (36.7)	171 (43.7)	66 (34.9)	91 (46)	82 (25.4)	59 (33.5)
Unstable	471 (36.9)	109 (27.9)	61 (32.3)	37 (18.7)	190 (58.8)	74 (42)
Income						
Lower than one minimum wage	553 (43.3)	174 (44.5)	76 (40.2)	111 (56.1)	147 (45.5)	45 (25.6)
One or two minimum wages	418 (32.7)	147 (37.6)	65 (34.4)	44 (22.2)	90 (27.9)	72 (40.9)
Two or three minimum wages	99 (7.8)	35 (9.0)	21 (11.1)	13 (6.6)	10 (3.1)	20 (11.4)
Three or more minimum wages	79 (6.2)	18 (4.6)	15 (7.9)	11 (5.6)	11 (3.4)	24 (13.6)
Gender-based discrimination						
No	187 (14.6)	48 (12.3)	23 (12.2)	34 (17.2)	49 (15.2)	33 (18.8)
Yes	1090 (85.4)	343 (87.7)	166 (87.8)	164 (82.8)	274 (84.8)	143 (81.2)
Verbal or psychological violence in the past 12 months						
No	666 (52.2)	196 (50.1)	81 (42.9)	106 (53.5)	194 (60.1)	89 (50.6)
Yes	611 (47.8)	195 (49.9)	108 (57.1)	92 (46.5)	129 (39.9)	87 (49.4)
Physical violence in the past 12 months						
No	1071 (83.9)	332 (84.9)	159 (84.1)	172 (86.9)	266 (82.4)	142 (80.7)
Yes	201 (15.7)	59 (15.1)	30 (15.9)	24 (12.1)	54 (16.7)	34 (19.3)
Sexual abuse in the past 12 months						
No	617 (48.3)	183 (46.8)	85 (45)	86 (43.4)	167 (51.7)	96 (54.5)
Yes	654 (51.2)	208 (53.2)	103 (54.5)	111 (56.1)	152 (47.1)	80 (45.5)
Sex labor throughout life						
No	334 (26.2)	88 (22.5)	53 (28.0)	53 (26.8)	94 (29.1)	46 (26.1)
Yes (at least once)	404 (31.6)	141 (36.1)	36 (19.0)	45 (22.7)	147 (45,5)	35 (19.9)
Sometimes	266 (20.8)	77 (19.7)	62 (32.8)	43 (21.7)	41 (12.7)	43 (24.4)
Full time	273 (21.4)	85 (21.7)	38 (20.1)	57 (28.8)	41 (12,7)	52 (29.5)
Steady partner in the past 6 months						
No	657 (51.4)	182 (46.5)	82 (43.4)	81 (40.9)	224 (69.3)	88 (50.0)
Yes	620 (48.6)	209 (53.5)	107 (56.6)	117 (59.1)	99 (30.7)	88 (50.0)
Casual partner in the past 6 months						
No	709 (55.5)	224 (57.3)	77 (40.7)	97 (49.0)	219 (67.8)	92 (52.3)
Yes	568 (44.5)	167 (42.7)	112 (59.3)	101 (51.0)	104 (32.2)	84 (47.7)
Commercial partner in the past 6 months						
No	766 (60.0)	237 (60.6)	89 (47.1)	101 (51.0)	252 (78.0)	87 (49.4)
Yes	511 (40.0)	154 (39.4)	100 (52.9)	97 (49.0)	71 (22.0)	89 (50.6)

TGW: transgender women and *travestis*.

Most of them reported having been discriminated during their lifetime (85.4%), almost half of them (47.8%) reported history of verbal or psychological violence in the past 12 months, and 15.7% (201) and 51.2% (654), of physical and sexual violence, respectively, in the past 12 months of the study. Besides, 73.8% (943) had done sex work at least once, almost half (48.6%) reported having had a steady partner in the past six months, and 44.5% (568) and 40.0% (511) reported having had casual and commercial partners, respectively, also in the past six months (Table 2).

The factors that were significantly associated with higher chances of HIV testing in the past 12 months were: having suffered verbal or psychological violence in the past 12 months (aOR 1.39; 95%CI 1.08–1.79) and having had a casual partner in the past six months (aOR 1.35; 95%CI 1.04–1.74). Being 35 years old or older significantly reduced the changes of HIV testing in the past 12 months (aOR 0.73; 95%CI 0.56–0.95) (Table 2).

For syphilis testing in the past 12 months, the factors that significantly increased the chances were: verbal or psychological violence in the past 12 months (aOR 1.53; 95%CI 1.19–1.98) and having had a commercial partner in the past 12 months (aOR 1.60; 95%CI 1.24–2.07) (Table 3).

**Table 3:** Estimation of aOR for associated factors for HIV and syphilis testing in the past 12 months among TGW in the TransOdara Project, 2020-2021.

Outcome	Variables	aOR	95%CI
HIV testing in the past 12 months^*^			
Age (years)			
Up to 34		1.00	
35 or older		0.73	0.56–0.95
Verbal or psychological violence in the past 12 months			
No		1.00	
Yes		1.39	1.08–1.79
Casual partner in the past 6 months			
No		1.00	
Yes		1.35	1.04–1.74
Syphilis testing in the past 12 months^†^			
Verbal or psychological violence in the past 12 months			
No		1.00	
Yes		1.53	1.19–1.98
Commercial partner in the past 12 months			
No		1.00	
Yes		1.60	1.24–2.07

* ICC 0.085

† ICC 0.098. TGW: transgender women and *travestis*.

For estimations of association for HBV and HVC testing in the past 12 months, having attended high school (aOR 1.50; 95%CI 1.09–2.06), higher education or more (aOR 2.21; 95%CI 1.12–4.34), having suffered verbal or psychological violence in the past 12 months (aOR 1.39; 95%CI 1.06–1.83) and having had a commercial partner in the past six months (aOR 1.39; 95%CI 1.04–1.85) significantly increased the chances of HBV testing in the past 12 months; whereas having attended high school (aOR 1.55; 95%CI 1.14–2.15) and having had a commercial partner in the past six months (aOR 1.71; 95%CI 1.29–2.27) were significantly associated with higher chances of HCV testing in the past six months (Table 4).

**Table 4:** Estimation of aOR for the associated factors for HBV and HCV testing in the past 12 months among TGW in the TransOdara Project, 2020–2021.

Outcomes	Variables	aOR	95%CI
HBV testing in the past 12 months^*^			
Schooling			
Elementary school		1.00	
High school		1.50	1.09–2.06
Higher education/more		2.21	1.12–4.34
Verbal or psychological violence in the past 12 months			
No		1.00	
Yes		1.39	1.06–1.83
Commercial partner in the past 6 months			
No		1.00	
Yes		1.39	1.04–1.85
HCV testing in the past 12 months^†^			
Schooling			
Elementary school		1.00	
High school		1.57	1.14–2.15
Higher education/more		1.88	0.97–3.69
Commercial partner in the past 6 months			
No		1.00	
Yes		1.71	1.29–2.27

* ICC 0.206

† ICC 0.206. HBV: hepatitis B; HCV: hepatitis C; TGW: transgender women and *travestis*.

## DISCUSSION

This study revealed that more than half of the TGW underwent a HIV or syphilis test in the past 12 months; however, less than half had tests for HBV or HCV in this period. Besides, there were factors such as schooling, income, sexual partners, sex work and violence, which were associated with testing, as already shown in the literature^20,28-34^.

The frequency of HIV testing in the past 12 months for TGW in this study was similar to that observed in other studies. An analysis carried out with TGW in Ho Chi Mihn, Vietnam, revealed a 59.3% frequency in the past year^32^. Another study conducted in Pattaya, Thailand, found 54.7% for testing in the past year among TGW^35^. In Brazil, a RDS analysis with TGW carried out in three cities found an even lower frequency (45.8%)^36^.

Until the moment when this study was carried out, there was no knowledge about other studies estimating the frequency of prior syphilis testing among TGW in Brazil. Considering that similar frequencies for HIV and syphilis testing were observed in the year before the study was carried out, it is possible that both tests had been taken simultaneously, given the recommendation established by the Ministry of Health for the conduction of STI testing in Brazil^37^.

Even if more than half of the participants had been tested for HIV or syphilis in the year before this study was conducted, this frequency is still low when compared to the testing recommendation recommended for this population. The Clinical Protocols and Therapeutic Guidelines (CPTGs) for comprehensive care addressed to people with STIs recommends that, for transgender women and *travestis*, HIV and syphilis screening should be performed at least twice a year^37^. Additionally, the updated strategy to face the HIV epidemic defined by the Joint United Nations Programme on HIV/AIDS (Unaids) in 2021 proposes that 95% of the people can be aware of their diagnosis, 95% can have access to treatment, and 95% have suppressed viral load^10,38^.

For prior HBV and HCV testing, a study carried out in Paris about access to rapid tests for HIV, HBC and HCV among men who had sex with other men (MSM), injectable drug users and transgender women and *travestis* also showed low testing frequency throughout life and in the past 12 months: for HBV, 49.3% had tests throughout life, and, of these, 49.3% had tests in the past 12 months; for HCV, 38.3% had tests throughout life, and, of these, 53.3% had tests in the past 12 months. Such low frequency can be explained by the lack of access of key populations to services of STI care and prevention^28^. Especially in the Brazilian context, the low prevalence of HBC and HCV among TGW can be considered to explain a less active orientation towards the screening of these STIs in this population^37^.

This study showed that older TGW had less chances of HIV testing in the past year. Maybe because they have experienced, for years, the lack of care by the health services in Brazil, the lack of specific health public policies addressed to the transgender population, besides the frequent cases of discrimination in the services coming from employees and users^39,40^. So, they might have internalized these experiences negatively, developing fear when it comes to searching for care, as well as some level of incredibility for the services^34,41-43^. It is necessary to mention that, despite the creation of trans-centered care strategies and policies throughout the years in Brazil, the practical reality of many TGW still is the lack of access and care, both in terms of prevention strategies and STI control and for health in a broader scenario^36,39,40,44,45^. Besides the lack of access, the conduction of a rapid test can also be affected by acceptability, which includes low cost, availability and time for the result or professional reception^46^. However, the acceptability to rapid testing was not verified in this study.

The report of suffering from verbal or psychological violence in the past year was also associated with more chances of testing. This finding is in accordance with other studies, which also reported the relationship between violence and infection by HIV. Exposure to violence might motivate testing among transgender women and *travestis*, given the knowledge about the increased risk and exposure for the transmission of this virus^20,33,47,48^. Besides, violence suffered by TGW can also be present in the context of sexual partners, especially when the test result is positive^29^.

Another significant factor regarding testing was the indication of a casual sex partner or commercial sex partner in the past six months, which was also related with the increasing chance of frequency in testing in the past 12 months. Such an effect may indicate knowledge and perception of risk for the participants, resulting in the adoption of preventive strategies among TGW with sexually active lives and casual or commercial partners. Such a practice enables to know the diagnosis soon and to adopt safe practices with their partners^32,49^. It is also important to mention that this practice may represent both more knowledge and the increasing search for care and prevention among TGW.

This study has some limitations. The cross-sectional design makes it difficult to establish a temporal relationship between the variables and, as well as in other studies with RDS, the existence of a selection bias, given the non-probability recruitment strategy and the homophily effect in the construction of the network^22^. Generally, these studies do not prevent the investigation of important information in populations that are hard to access for conventional recruitment methodologies, as well as strategies such as the selection of random characteristics of the seeds and stochastic network adjustment tend to reduce selection bias. The COVID-19 pandemic may have added some limitations, considering it impacted conduction in different sites, including recruitment, which as adjusted afterwards. Therefore, the use of a data modelling methodology that would examine these effects in the level of each site was thought of, considering the possibility that these differences existed.

It is clear that the stigma that is present in society and the difficulties in access to health services and care, as well as gender-based discrimination, are barriers for access to health in general, and STIs in particular^36,39,40,45^. Throughout the years, efforts have been made by the organized civil society to ensure the rights of the transgender population, as well as to create a national comprehensive health policy for lesbians, gays, bisexuals, transgender and *travestis* ^50^. Still, it is necessary to put in practice strategies that are, in fact, accessible, comprehensive and longitudinal, which meet the specificities of Brazilian TGW, especially regarding rapid testing for STIs. The effective decentralization of the STI prevention policy and the fight against the stigma and discrimination are key points for such an increase.
