# Supplementary material for: Factors associated with prior testing for HIV, Syphilis, and Hepatitis B and C among transgender women and travestis in Brazil
Source: Rev Bras Epidemiol. 2024 Aug 19;27(Suppl 1):e240008.supl.1. doi: 10.1590/1980-549720240008.supl.1 (PMC11338533; doi:10.1590/1980-549720240008.supl.1)
Supplement: Translation

# Translation: Fatores associados à realização de testagem prévia para HIV, sífilis e hepatites B e C entre mulheres trans e travestis no Brasil

**DOI:** 10.1590/1980-549720240008.supl.1.2

**Authors:** Beo Oliveira Leite, Inês Dourado, Laio Magno, Sandro Sperandei, Carla Gianna Luppi, Maria Amelia de Sousa Mascena Veras

**Affiliations:** I Universidade Federal da Bahia, Instituto de Saúde Coletiva – Salvador (BA), Brasil.; II Universidade do Estado da Bahia, Departamento de Ciências da Vida – Salvador (BA), Brasil.; III Western Sydney University, Translational Health Research Institute – Penrith, Astralia.; IV Universidade Federal de São Paulo, Escola Paulista de Medicina, Departamento de Medicina Preventiva – São Paulo (SP), Brasil.; V Santa Casa de São Paulo, Faculdade de Ciências Médicas – São Paulo (SP), Brasil.

**Keywords:** Pessoas transgênero, Testes sorológicos, HIV, Sífilis, Hepatite B, Hepatite C.

## Abstract

**Objetivo:**

Investigar a realização de testagem prévia de HIV, sífilis, hepatites B (HBV) e C (HCV) entre mulheres trans e travestis (MTT) em cinco cidades brasileiras e identificar fatores associados à testagem.

**Métodos:**

Trata-se de um estudo de corte transversal, com recrutamento de MTT através do *respondent-driven sampling* (Projeto TransOdara). A variável de desfecho investigada foi realização de testagem prévia HIV, Sífilis, HBV e HCV nos últimos 12 meses. A associação entre fatores sociodemográficos e comportamentais com o desfecho foi analisada usando modelo de regressão logística binomial com efeitos mistos. Estimou-se *odds ratio* ajustada (aOR) e intervalos de confiança a 95% (IC95%).

**Resultados:**

As proporções de pessoas com realização prévia de testagem foram: 56,3% para HIV, 58,0% para sífilis, 42,1% para HBV e 44,7% para HCV. Observaram-se associação negativa da testagem prévia com idade de 35 anos ou mais e associação positiva com ter ensino médio, ter sofrido violência verbal ou psicológica nos últimos 12 meses e ter tido parceiro comercial ou casual nos últimos seis meses.

**Conclusão:**

Verificou-se baixa frequência de testagem nos últimos 12 meses anteriores ao estudo para HIV, Sífilis, HBV e HCV em comparação às orientações estipuladas pelo Ministério da Saúde. A ampliação do acesso e vinculação aos serviços de atenção e prevenção para as MTT é uma estratégia essencial para a redução da cadeia de transmissão do HIV e outras infecções sexualmente transmissíveis.

## INTRODUÇÃO

Estima-se que em todo o mundo cerca de um milhão de novas infecções sexualmente transmissíveis (ISTs) ocorrem diariamente na população em geral. A grande maioria costuma se desenvolver de forma assintomática e, quando não diagnosticada e tratada em tempo hábil, pode impactar negativamente a saúde individual, bem como o aumento de gastos diretos em saúde^1^. Portanto, a testagem oportuna é essencial para ações de vigilância epidemiológica, ou seja, identificação e tratamento de novos casos e, consequentemente, interrupção da cadeia de transmissão.

Entre as diversas estratégias propostas pela Organização Mundial da Saúde (OMS) para conter o avanço da ocorrência das ISTs, a testagem múltipla integrada consiste na investigação de infecções através da oferta conjunta de diversos testes rápidos em uma mesma visita (e.g., HIV, sífilis, hepatites virais etc.) para diagnóstico em tempo hábil, vinculação dos indivíduos com o serviço, tratamento imediato e promoção de ações de prevenção. Para que tais ações ocorram, a expansão de acesso a testagens para diferentes ISTs deve ser uma prioridade alinhada com ações de prevenção e promoção da saúde^1^. Entretanto se observam falhas no acesso e perda de oportunidades para testagem, que é desproporcionalmente maior entre os grupos mais vulneráveis a essas infecções^2,3^.

Para esses grupos populacionais, também conhecidos como populações-chave (i.e., usuários de drogas injetáveis, prisioneiros e outras pessoas encarceradas, trabalhadores do sexo, homens que fazem sexo com outros homens e pessoas trans), é observado maior risco e vulnerabilidade de infeção por ISTs, especialmente quando se verificam maiores prevalências dessas infecções quando comparado com a população em geral^3-5^. Além disso, observa-se menor acesso aos serviços de saúde voltados a prevenção, testagem e tratamento^3-10^.

Entre as pessoas trans, as mulheres trans e travestis (MTT) em todo mundo são desproporcionalmente mais afetadas por ISTs. A prevalência global de HIV estimada para mulheres trans é de 19,9%^11^; para sífilis, entre 1,4 e 50,4%^12^; e as prevalências estimadas de hepatites B (HBV) e C (HCV) são de 5,0 e 6,0% respectivamente^13^. No Brasil, as prevalências são desproporcionalmente maiores em comparação com a população em geral^4,14^: HIV variando de 14,3 a 40,9%^11,13^; sífilis, de 28,9 a 61,7%^12-17^; HBV, de 0,7 a 12,3%^16,18,19^; e HCV, de 0,8 a 1,4%^13,18,19^.

O risco e a vulnerabilidade acrescidos para ISTs entre as MTT podem ser explicados por múltiplos fatores: comportamentais, como sexo sem uso de preservativo; programáticos, como o menor acesso aos serviços de saúde; e sociais, como estigma e a discriminação de gênero aos quais essa população é frequentemente submetida^20,21^. Além do mais, a compreensão da interseção entre estes múltiplos fatores é essencial para avaliar diferentes graus de risco para o HIV como um fenômeno complexo. A investigação de tais fatores é fundamental para ampliar o conhecimento sobre o conjunto de aspectos que caracterizam a vulnerabilidade dessas populações e promover a melhoria do acesso à testagem para ISTs. O objetivo deste estudo foi investigar a realização de testagem prévia de HIV, sífilis, hepatites B (HBV) e C (HCV) entre MTT em cinco cidades brasileiras e identificar fatores associados à testagem.

## MÉTODOS

### Desenho, local e população do estudo

Esta análise integra o estudo TransOdara, intitulado “Estudo de prevalência da sífilis e outras infecções sexualmente transmissíveis entre travestis e mulheres transexuais no Brasil: cuidado e prevenção”, desenvolvido entre dezembro 2019 e julho 2021 em cinco capitais (Campo Grande, Manaus, Porto Alegre, Salvador e São Paulo). O estudo foi coordenado pela Faculdade de Ciências Médicas da Santa Casa de São Paulo, em colaboração com o Centro de Referência e Treinamento em DST/Aids (CRT DST/Aids), Universidade Federal do Rio Grande do Sul (UFRGS), Universidade Federal da Bahia (UFBA), Universidade Federal do Mato Grosso do Sul (UFMS), Fundação Leônidas e Maria Deane (Fiocruz – Manaus), Secretaria Municipal de Saúde de Porto Alegre (SMS-POA), Instituto Adolfo Lutz (IAL) e Universidade Federal de Ciências da Saúde de Porto Alegre (UFCSPA).

A população de estudo foi composta por pessoas que se auto identificaram como mulheres trans e travestis (MTT). Travesti é um termo etnocultural latino-americano utilizado por algumas pessoas trans como uma identidade feminina.

As MTT elegíveis foram aquelas que apresentaram os seguintes critérios:

1. Ter com idade ≥18 anos;
2. Ter sido atribuída atribuído o sexo masculino ao nascer e a autoidentificação atual com uma identidade de gênero feminina;
3. Ser residentes na área metropolitana de uma das cidades do estudo; e
4. Ter um cupom válido para ingresso na pesquisa.

### Coleta de dados e amostragem

A técnica amostral utilizada foi *respondent driven sampling* (RDS), indicada para populações consideradas de difícil acesso, nas quais o recrutamento tem maior alcance quando feito por meio dos próprios participantes, e que utiliza de métodos estatísticos para ajustar o recrutamento realizado por pares em uma rede de contato^22-24^.

As primeiras participantes, denominados “sementes”, foram selecionadas de forma não aleatória pelos pesquisadores após pesquisa qualitativa formativa, de modo a representar a heterogeneidade da população de MTT, segundo condições demográficas e socioeconômicas. Cada uma das sementes recebia até seis cupons para recrutar outras mulheres trans e travestis de sua rede de contatos, e assim sucessivamente. Cada participante recebia dois tipos de ressarcimento financeiro: o primário pela participação no estudo e o secundário por cada MTT que recrutasse.

O estudo ocorreu nos centros de pesquisa de cada uma das instituições colaboradoras dos cinco sítios. As participantes eram informadas sobre todos os procedimentos realizados na pesquisa, recebiam materiais educativos, preservativos e gel lubrificante, confirmavam a elegibilidade para o estudo e assinavam o termo de consentimento livre e esclarecido (TCLE). Após a assinatura do TCLE, as participantes eram encaminhadas para responder um questionário sociocomportamental com um(a) entrevistador(a) previamente treinado. O questionário foi dividido em questões com os seguintes temas: variáveis socioeconômicas e demográficas; acesso e uso de serviços de saúde; utilização de hormônios e modificações corporais; conhecimento sobre ISTs, testagem e comportamento sexual; estigma, discriminação e violência; uso de álcool e outras drogas. Para mais informações, ver Veras et al.^25^.

### Variáveis do estudo

*Desfechos:* realização de quaisquer tipos de testagem para IST nos últimos 12 meses:

1. Testagem de HIV nos últimos 12 meses (não vs. sim);
2. Testagem de Sífilis nos últimos 12 meses (não vs. sim);
3. Testagem de HBV nos últimos 12 meses (não vs. sim);
4. Testagem de HCV nos últimos 12 meses (não vs. sim).

*Variáveis preditoras:* mudou o nome de registro (não vs. sim); idade (até 35 anos vs. mais de 35 anos); raça/cor de pele (branco, preto/pardo e outro); escolaridade (ensino fundamental, ensino médio e ensino superior/maior); tipo de moradia (própria, aluguel e instável [ex.: temporária ou provisória que não se caracterizam como aluguel]); renda (menor que um salário mínimo, um a dois salários mínimos, dois a três salários mínimos e três ou mais salários mínimos); sofreu discriminação de gênero (não vs. sim); histórico de violência verbal ou psicológica nos últimos 12 meses (não vs. sim); histórico de violência física nos últimos 12 meses (não vs. sim); histórico de abuso sexual nos últimos 12 meses (não vs. sim); trabalho sexual na vida (não vs. sim); parceiro fixo nos últimos seis meses (não vs. sim); parceiro casual nos últimos seis meses (não vs. sim); parceiro comercial nos últimos seis meses (não vs. sim).

### Análise dos dados

As variáveis foram descritas através das frequências absolutas e relativas. A associação das variáveis independentes e cada desfecho foi avaliada utilizando modelos de regressão logísticos mistos com interceptos aleatórios para as cidades de coleta dos dados (Campo Grande, Manaus, Porto Alegre, Salvador e São Paulo, representado as cinco macrorregiões brasileiras). Os pesos provenientes da amostragem RDS não foram utilizados, seguindo a recomendação de Sperandei et al.^26^. O modelo múltiplo final foi selecionado utilizando o pacote StatisticalModels para linguagem R, através de um *backward stepwise*, iniciando com o modelo completo com todas as variáveis e retirando variáveis uma a uma, até que o menor modelo significativo seja alcançado. A máxima verossimilhança (ML) foi utilizada na seleção dos modelos, e a máxima verossimilhança restrita (REML) foi utilizada no ajuste do modelo final. Os resultados dos modelos logísticos foram apresentados em razão de chances (*odds ratio* – OR) e seus respectivos intervalos de confiança para 95%. As variabilidades de cada um dos desfechos foram estimadas através do coeficiente de correlação intraclasse (ICC). Todas as análises foram realizadas em R versão 4.2.3^27^.

### Aspectos éticos

O projeto foi aprovado pelo Comitê de Ética em Pesquisa (CEP) da Santa Casa de Misericórdia de São Paulo (CAAE 05585518.7.0000.5479 - Nº parecer: 3.126.815 - 30/01/2019), assim como pelas demais instituições participantes.

## RESULTADOS

Das 1.317 MTT recrutadas, 1.277 responderam às perguntas sobre testagem prévia para ISTs, classificadas como desfecho e foram incluídas nesta análise. Dessas, 55,3% (n=706; IC95% 52,5–58,4) realizaram testagem para HIV nos últimos 12 meses; 56,1% (n=716; IC95% 53,3–58,8), para sífilis; 40,0% (n=511; IC95% 37,3–42,8), para HBV; e 42,6% (n=544; IC95% 39,9–45,4), para HCV (Tabela 1). Do total, 30,6% (n=391) residiam ou trabalhavam em São Paulo; 25,3% (n=323), em Manaus; 15,5% (n=198), em Salvador; 14,8% (n=189), em Porto Alegre; e 13,8% (n=176), em Campo Grande. A maioria delas ainda não havia mudado o nome de registro (70,6%), possuíam até 34 anos de idade (63,0%), eram pretas ou pardas (70,1%), possuíam nível de escolaridade até o ensino médio (69,9%), moravam de aluguel (36,7%) ou moradia instável (36,9%) e possuíam renda menor que um salário mínimo (43,3%) (Tabela 2).

**Tabela 1:** Descrição sociodemográfica e comportamental das MTT do Projeto TransOdara, 2020–2021.

Variáveis (n=1.277)	Cidade	n (%)	IC95%
Testagem de HIV nos últimos 12 meses	São Paulo	308 (76,4)	71,6–81,2
	Porto Alegre	84 (43,8)	33,0–54,6
	Salvador	101 (50,0)	40,1–59,9
	Manaus	141 (41,6)	33,4–49,8
	Campo Grande	89 (49,2)	38,7–59,7
	General	706 (55,3)	52,5–58,4
Testagem de sífilis nos últimos 12 meses	São Paulo	316 (78,4)	73,8–83,0
	Porto Alegre	88 (45,8)	35,2–56,4
	Salvador	96 (47,5)	37,4–57,6
	Manaus	146 (43,1)	35,0–51,2
	Campo Grande	88 (48,6)	38,0–59,2
	General	716 (56,1)	53,3–58,8
Testagem de HBV nos últimos 12 meses	São Paulo	301 (74,7)	69,8–79,6
	Porto Alegre	67 (34,6)	23,3–46,5
	Salvador	49 (24,3)	12,0–36,6
	Manaus	48 (14,2)	4,1–24,3
	Campo Grande	58 (32,0)	19,7–44,3
	General	511 (40,0)	37,3–42,8
Testagem de HCV nos últimos 12 meses	São Paulo	314 (77,9)	73,3–82,5
	Porto Alegre	73 (38,0)	26,7–49,3
	Salvador	54 (26,7)	14,6–38,8
	Manaus	61 (18,0)	8,2–27,8
	Campo Grande	53 (29,3)	16,8–41,8
	Geral	544 (42,6)	39,9–45,4

MTT: mulheres trans e travestis; HBV: hepatites B; HCV: hepatites C.

**Tabela 2:** Descrição sociodemográfica e comportamental das MTT do Projeto TransOdara, 2020–2021.

Variáveis (n=1277)	n (%)	São Paulo	Porto Alegre	Salvador	Manaus	Campo Grande
Mudou o nome de registro						
Não	902 (70,6)	220 (56,3)	107 (56,6)	146 (73,7)	296 (91,6)	133 (75,6)
Sim	374 (29,3)	170 (43,5)	82 (43,4)	52 (26,3)	27 (8,4)	43 (24,4)
Idade (anos)						
Até 34	804 (63,0)	214 (54,7)	115 (60,8)	141 (71,2)	218 (67,5)	116 (65,9)
35 ou mais	473 (37,0)	177 (45,3)	74 (39,2)	57 (28,8)	105 (32,5)	60 (34,1)
Raça/cor de pele						
Branca	328 (25,7)	105 (26,9)	90 (47,6)	21 (10,6)	59 (18,3)	53 (30,1)
Preta/parda	895 (70,1)	274 (70,1)	95 (50,3)	171 (86,4)	242 (74,9)	113 (64,2)
Outra	44 (3,4)	11 (2,8)	4 (2,1)	6 (3)	20 (6,2)	3 (1,7)
Escolaridade						
Ensino fundamental	323 (25,3)	95 (24,3)	52 (27,5)	53 (26,8)	75 (23,2)	48 (27,3)
Ensino médio	892 (69,9)	274 (70,1)	126 (66,7)	140 (70,7)	230 (71,2)	122 (69,3)
Ensino superior ou mais	62 (4,9)	22 (5,6)	11 (5,8)	5 (2,5)	18 (5,6)	6 (3,4)
Moradia						
Própria	337 (26,4)	111 (28,4)	62 (32,8)	70 (35,4)	51 (15,8)	43 (24,4)
Aluguel	469 (36,7)	171 (43,7)	66 (34,9)	91 (46)	82 (25,4)	59 (33,5)
Instável	471 (36,9)	109 (27,9)	61 (32,3)	37 (18,7)	190 (58,8)	74 (42)
Renda						
Menor que um salário mínimo	553 (43,3)	174 (44,5)	76 (40,2)	111 (56,1)	147 (45,5)	45 (25,6)
Um ou dois salários mínimos	418 (32,7)	147 (37,6)	65 (34,4)	44 (22,2)	90 (27,9)	72 (40,9)
Dois ou três salários mínimos	99 (7,8)	35 (9,0)	21 (11,1)	13 (6,6)	10 (3,1)	20 (11,4)
Três ou mais salários mínimos	79 (6,2)	18 (4,6)	15 (7,9)	11 (5,6)	11 (3,4)	24 (13,6)
Discriminação de gênero						
Não	187 (14,6)	48 (12,3)	23 (12,2)	34 (17,2)	49 (15,2)	33 (18,8)
Sim	1090 (85,4)	343 (87,7)	166 (87,8)	164 (82,8)	274 (84,8)	143 (81,2)
Violência verbal ou psicológica nos últimos 12 meses						
Não	666 (52,2)	196 (50,1)	81 (42,9)	106 (53,5)	194 (60,1)	89 (50,6)
Sim	611 (47,8)	195 (49,9)	108 (57,1)	92 (46,5)	129 (39,9)	87 (49,4)
Violência física nos últimos 12 meses						
Não	1071 (83,9)	332 (84,9)	159 (84,1)	172 (86,9)	266 (82,4)	142 (80,7)
Sim	201 (15,7)	59 (15,1)	30 (15,9)	24 (12,1)	54 (16,7)	34 (19,3)
Abuso sexual nos últimos 12 meses						
Não	617 (48,3)	183 (46,8)	85 (45)	86 (43,4)	167 (51,7)	96 (54,5)
Sim	654 (51,2)	208 (53,2)	103 (54,5)	111 (56,1)	152 (47,1)	80 (45,5)
Trabalho sexual na vida						
Não	334 (26,2)	88 (22,5)	53 (28,0)	53 (26,8)	94 (29,1)	46 (26,1)
Já trabalhou	404 (31,6)	141 (36,1)	36 (19,0)	45 (22,7)	147 (45,5)	35 (19,9)
Trabalha às vezes	266 (20,8)	77 (19,7)	62 (32,8)	43 (21,7)	41 (12,7)	43 (24,4)
Trabalha em tempo integral	273 (21,4)	85 (21,7)	38 (20,1)	57 (28,8)	41 (12,7)	52 (29,5)
Parceiro fixo nos últimos 6 meses						
Não	657 (51,4)	182 (46,5)	82 (43,4)	81 (40,9)	224 (69,3)	88 (50,0)
Sim	620 (48,6)	209 (53,5)	107 (56,6)	117 (59,1)	99 (30,7)	88 (50,0)
Parceiro casual nos últimos 6 meses						
Não	709 (55,5)	224 (57,3)	77 (40,7)	97 (49,0)	219 (67,8)	92 (52,3)
Sim	568 (44,5)	167 (42,7)	112 (59,3)	101 (51,0)	104 (32,2)	84 (47,7)
Parceiro comercial nos últimos 6 meses						
Não	766 (60,0)	237 (60,6)	89 (47,1)	101 (51,0)	252 (78,0)	87 (49,4)
Sim	511 (40,0)	154 (39,4)	100 (52,9)	97 (49,0)	71 (22,0)	89 (50,6)

MTT: mulheres trans e travestis.

Maior parte delas relatou já ter sofrido discriminação alguma vez na vida (85,4%), quase metade (47,8%) relatou histórico de violência verbal ou psicológica nos últimos 12 meses e 15,7% (201) e 51,2% (654), de violência física e sexual respectivamente nos últimos 12 meses da realização da pesquisa. Além disso, 73,8% (943) já havia feito trabalho sexual alguma vez na vida, quase metade (48,6%) relatou ter tido parceiro fixo nos últimos seis meses e 44,5% (568) e 40,0% (511), relataram ter tido parceiros casuais e comerciais respectivamente, também nos últimos seis meses (Tabela 2).

Os fatores que se associaram significativamente a uma maior chance de realização de testagem para HIV nos últimos 12 meses foram: ter sofrido violência verbal ou psicológica nos últimos 12 meses (aOR 1,39; IC95% 1,08–1,79) e ter tido parceiro casual nos últimos seis meses (aOR 1,35; IC95% 1,04–1,74). A idade de 35 anos ou mais reduziu significativamente a chance de realização de testagem para HIV nos últimos 12 meses (aOR 0,73; IC95% 0,56–0,95) (Tabela 2).

Para a realização de testagem de sífilis nos últimos 12 meses, os fatores que aumentaram significativamente a chance foram: violência verbal ou psicológica nos últimos 12 meses (aOR 1,53; IC95% 1,19–1,98) e ter tido parceiro comercial nos últimos 12 meses (aOR 1,60; IC95% 1,24–2,07) (Tabela 3).

**Tabela 3:** Estimativa da aOR para os fatores associados para testagem de HIV e sífilis nos últimos 12 meses entre as MTT do Projeto TransOdara, 2020-2021.

Desfecho	Variáveis	aOR	IC95%
Testagem de HIV nos últimos 12 meses^*^			
Idade (anos)			
Até 34		1,00	
35 ou mais		0,73	0,56–0,95
Violência verbal ou psicológica nos últimos 12 meses			
Não		1,00	
Sim		1,39	1,08–1,79
Parceiro casual nos últimos 6 meses			
Não		1,00	
Sim		1,35	1,04–1,74
Testagem de sífilis nos últimos 12 meses^†^			
Violência verbal ou psicológica nos últimos 12 meses			
Não		1,00	
Sim		1,53	1,19–1,98
Parceiro comercial nos últimos 12 meses			
Não		1,00	
Sim		1,60	1,24–2,07

* ICC 0,085

† ICC 0,098. MTT: mulheres trans e travestis.

Para as estimativas de associação para realização de testagem HBV e HCV nos últimos 12 meses, ter ensino médio (aOR 1,50; IC95% 1,09–2,06), ter ensino superior ou maior (aOR 2,21; IC95% 1,12–4,34), ter sofrido violência verbal ou psicológica nos últimos 12 meses (aOR 1,39; IC95% 1,06–1,83) e ter tido parceiro comercial nos últimos 12 meses (aOR 1,39; IC95% 1,04–1,85) aumentaram significativamente a chance de ter realizado teste para HBV nos últimos 12 meses; enquanto ter ensino médio (aOR 1,55; IC95% 1,14–2,15) e ter tido parceiro comercial nos últimos seis meses (aOR 1,71; IC95% 1,29–2,27) estiveram associados significativamente a uma maior chance de realização de testagem para HCV nos últimos seis meses (Tabela 4).

**Tabela 4:** Estimativa da aOR para os fatores associados para testagem para testagem de HBV e HCV nos últimos 12 meses entre as MTT do estudo TransOdara, 2020–2021.

Desfechos	Variáveis	aOR	IC95%
Testagem de HBV nos últimos 12 meses^*^			
Escolaridade			
Ensino fundamental		1,00	
Ensino médio		1,50	1,09–2,06
Ensino superior/maior		2,21	1,12–4,34
Violência verbal ou psicológica nos últimos 12 meses			
Não		1,00	
Sim		1,39	1,06–1,83
Parceiro comercial nos últimos 6 meses			
Não		1,00	
Sim		1,39	1,04–1,85
Testagem de HCV nos últimos 12 meses^†^			
Escolaridade			
Ensino fundamental		1,00	
Ensino médio		1,57	1,14–2,15
Ensino superior/maior		1,88	0,97–3,69
Parceiro comercial nos últimos 6 meses			
Não		1,00	
Sim		1,71	1,29–2,27

* ICC 0,206

† ICC 0,206. HBV: hepatites B; HCV: hepatites C; MTT: mulheres trans e travestis.

## DISCUSSÃO

Este estudo revelou que mais da metade das MTT realizou algum teste para HIV ou sífilis nos últimos 12 meses, entretanto menos da metade chegou a realizar testes para HBV ou HCV nesse mesmo período. Além do mais, observou-se que fatores como escolaridade, renda, parcerias sexuais, trabalho sexual e violência foram associados com a realização das testagens, assim como já mostrado na literatura^20,28-34^.

A frequência de realização da testagem para HIV nos últimos 12 meses para MTT encontrada neste estudo foi similar à observada em outros estudos. Um estudo realizado com MTT em Ho Chi Minh, Vietnã, revelou uma frequência de 59,3% no último ano^32^. Já outro estudo realizado em Pattaya, Tailândia, encontrou 54,7% de testagem no último ano entre MTT^35^. Já no Brasil, um estudo de RDS com MTT realizado em três cidades encontrou uma frequência ainda menor (45,8%)^36^.

Até o momento da realização deste estudo, não se tem conhecimento de outros estudos que estimassem a frequência de realização prévia de testagem de sífilis entre MTT no Brasil. Visto que se observaram frequências semelhantes de realização para testagem de HIV e sífilis no último ano anterior à realização da pesquisa, é possível que ambos os testes tenham sido realizados concomitantemente, dada a recomendação estabelecida pelo Ministério da Saúde para realização de testagem para ISTs no Brasil^37^.

Ainda que mais da metade das participantes tenham realizado a testagem para HIV ou sífilis no ano anterior à realização desta pesquisa, essa frequência ainda é baixa quando comparada com a recomendação de testagem prevista para essa população. O protocolo de diretrizes clínicas e terapêuticas (PCDT) para atenção integral às pessoas com ISTs recomenda que, para mulheres trans e travestis, o rastreio de HIV e sífilis seja realizado ao menos duas vezes ao ano^37^. Adicionalmente, a atualização da estratégia de enfrentamento da epidemia do HIV definida pelo Programa Conjunto das Nações Unidas sobre HIV/Aids (Unaids) em 2021 propõe que 95% das pessoas conheçam seu diagnóstico, 95% tenham acesso ao tratamento e 95% tenham carga viral suprimida^10,38^.

Para a testagem prévia para HBV e HCV, um estudo realizado em Paris sobre acesso aos testes rápidos para HIV, HBC e HCV entre homens que faziam sexo com outros homens (HSH), usuários de drogas injetáveis e mulheres trans e travestis revelou também uma baixa frequência de testagem na vida e nos últimos 12 meses: para HBV, 49,3% fizeram testagem na vida e, destas, 49,3% fizeram nos últimos 12 meses; já para HCV, 38,3% já fizeram testagem na vida e, destas, 53,3% fizeram nos últimos 12 meses. Essa baixa frequência pode ser explicada pela falta de acesso das populações-chave para os serviços de atenção e prevenção às IST^28^. Especialmente no contexto brasileiro, pode-se levar em conta a baixa prevalência de HBV e HCV entre as MTT, o que explica uma orientação menos ativa para rastreio dessas ISTs nessa população^37^.

Este estudo revelou que as MTT mais velhas tiveram menor chance de realização de testagem para HIV no último ano. Talvez por terem experenciado, durante anos, a falta de cuidado pelos serviços de saúde no Brasil, a falta de políticas públicas de saúde específicas voltadas à população trans, além das frequentes ocasiões de discriminação nos serviços por parte dos profissionais e usuários^39,40^, podem ter internalizado negativamente tais experiências, desenvolvendo um medo pela procura de assistência, assim como um certo grau de falta de credibilidade dos serviços^34,41-43^. Faz-se necessário salientar que, apesar da criação de estratégias e políticas de atenção trans centradas ao longo dos últimos anos no Brasil, a realidade prática de muitas MTT ainda é a falta de acesso e assistência tanto para estratégias de prevenção e controle das ISTs como para a saúde de uma forma ampliada^36,39,40,44,45^. Além da falta de acesso, a realização do teste rápido também pode ser afetada pela aceitabilidade, o que inclui baixo custo, disponibilidade e tempo para o resultado ou o acolhimento pelo profissional^46^. Entretanto a aceitabilidade à testagem rápida não foi aferida neste estudo.

O relato de sofrimento de violência verbal ou psicológica no último ano também esteve associado com a maior chance de testagem. Esse achado está em consonância com outros estudos, que também reportaram a relação entre violência e infecção pelo HIV. A exposição à violência poderia motivar a realização de testagem entre mulheres trans e travestis, dado o conhecimento sobre o risco e exposição aumentados para a transmissão desse vírus^20,33,47,48^. Além do mais, a violência sofrida pelas MTT também pode estar presente no contexto das parcerias sexuais, especialmente quando o resultado do teste é positivo^29^.

Outro fator significante na realização de testagens foi a indicação de parceria sexual casual ou comercial nos últimos seis meses, as quais também estiveram relacionadas com o aumento da chance de frequência das testagens nos últimos 12 meses. Tal efeito pode indicar conhecimento e percepção de risco das participantes, resultando na adoção de estratégias preventivas entre as MTT com vida sexualmente ativa e parcerias casuais ou comerciais. Tal prática possibilita o conhecimento do diagnóstico com brevidade e a adoção de práticas seguras entre seus parceiros^32,49^. É também importante ressaltar que a adoção dessa prática pode representar tanto o aumento do conhecimento como o aumento da procura pelo cuidado e pela prevenção entre as MTT.

Este estudo possuiu algumas limitações. O desenho transversal dificulta a possibilidade de estabelecimentos de uma relação temporal entre as variáveis e, assim como em outros estudos que utilizam RDS, a existência de viés seleção, dada a estratégia de recrutamento não probabilística e efeito de homofilia na construção da rede^22^. De uma forma geral, tais estudos não impedem a investigação de importantes informações em populações de difícil acesso para as metodologias de recrutamento convencionais, assim como estratégias como a seleção de características aleatórias das sementes e ajuste estocástico da rede tendem a reduzir o viés de seleção. A ocorrência da pandemia do COVID-19 pode ter adicionado limitações, visto que impactou a realização dos campos nos diferentes sítios, incluindo recrutamento, o que foi posteriormente ajustado. Dessa forma, considerou-se o uso de uma metodologia de modelagem dos dados que examinasse tais efeitos em nível de cada sítio, levando em conta a possibilidade de existência destas diferenças.

É evidente que o estigma presente na sociedade e as dificuldades de acesso aos serviços e cuidados de saúde, bem como a discriminação de gênero, são barreiras para o acesso aos cuidados em saúde em geral e às IST em particular^36,39,40,45^. Ao longo dos anos, esforços têm sido realizados pela sociedade civil organizada na garantia de direitos para população trans, assim como na criação da política nacional de saúde integral para lésbicas, *gays*, bissexuais, travestis e transexuais (PNSILGBT)^50^. Ainda assim, faz-se necessário pôr em prática estratégias que sejam, de fato, acessíveis, integrais e longitudinais e atendam às especificidades das MTT brasileiras, especialmente no que se diz respeito à testagem rápida para as ISTs. A efetiva descentralização da política de prevenção às ISTs e o combate ao estigma e à discriminação são pontos-chave para essa ampliação.
